# Line-Field Confocal Optical Coherence Tomography: Is One Hour of Training Sufficient for Diagnosing Basal Cell Carcinoma?

**DOI:** 10.3390/cancers17050826

**Published:** 2025-02-27

**Authors:** Elisa Cinotti, Martina D’Onghia, Alessandra Cartocci, Sofia Lo Conte, Lorenzo Barbarossa, Clara Tavernier, Giovanni Rubegni, Linda Tognetti, Mariano Suppa, Pietro Rubegni

**Affiliations:** 1 Dermatology Unit, Department of Medical, Surgical and Neurological Sciences, University of Siena, 53100 Siena, Italy; elisa.cinotti@unisi.it (E.C.); alessandra.cartocci@dbm.unisi.it (A.C.); sofia.loconte@student.unisi.it (S.L.C.); lorenzobarbarossa1993@gmail.com (L.B.); linda.tognetti@dbm.unisi.it (L.T.); pietro.rubegni@gmail.com (P.R.); 2 UOC Neuroradiologia Diagnostica e Terapeutica, AOU Senese, 53100 Siena, Italy; 3 DAMAE Medical, 75013 Paris, France; clara@damae-medical.com; 4 Department of Clinical Medicine and Immunological Sciences, Section of Ophthalmology, University of Siena, 53100 Siena, Italy; giovannirubegni@gmail.com; 5 Department of Dermatology, Hôpital Erasme, HUB, Université Libre de Bruxelles, 1050 Bruxelles, Belgium; dr.marianosuppa@gmail.com

**Keywords:** basal cell carcinoma, LC-OCT, non-invasive, imaging, training

## Abstract

**Background/Objectives:** Basal cell carcinoma (BCC) is the most common malignancy, characterised by local invasiveness and the potential for tissue destruction. Diagnosing BCC can be challenging, particularly for less experienced dermatologists. Line-field confocal optical coherence tomography (LC-OCT), a new noninvasive optical technique, has become increasingly useful in clinical practice, allowing in vivo imaging at cellular resolution. This study aimed to evaluate the impact of a 1 h structured training session on the ability of dermatology residents to diagnose BCC using LC-OCT. **Methods:** Eight dermatology residents with different levels of LC-OCT experience (no experienced and low experience in LC-OCT) participated alongside an expert dermatologist as a benchmark. Overall, participants evaluated 40 histopathologically confirmed lesions (20 BCC, 20 non-BCC) before and after training, with and without the inclusion of dermoscopic images. **Results:** We observed a significant improvement in diagnostic accuracy post-training, especially among inexperienced users, with a 20% reduction in false negatives and a 35% reduction in false positives. When the dermoscopic images were included, further improvements were noted, with the accuracy increasing by an additional 5%. The overall diagnostic rate for inexperienced readers increased from 48% to 76% after training and to 81% with the addition of dermoscopic images in the course. **Conclusions:** Our study highlights the effectiveness of short, targeted training in enhancing the diagnostic utility of LC-OCT, emphasising its potential for broader clinical adoption to improve BCC detection.

## Simple Summary

Basal cell carcinoma (BCC), the most common type of skin cancer, can be difficult to diagnose due to its resemblance to other benign and malignant skin lesions. Advanced non-invasive imaging techniques, such as line-field confocal optical coherence tomography (LC-OCT), offer highly detailed virtual skin biopsies, potentially enhancing diagnostic precision. This study investigated whether a 1 h training session could enhance dermatology residents’ ability to differentiate BCC from other lesions using LC-OCT. Our findings demonstrate that one hour of training significantly improves diagnostic performance, particularly among inexperienced users. These results highlight LC-OCT’s potential as a user-friendly and effective tool for diagnosing skin cancer, emphasising the importance of tailored training programs to optimise its clinical application.

## 1. Introduction

Basal cell carcinoma (BCC) is the most frequent skin malignancy, and its incidence is increasing globally [1,2]. While BCC usually grows slowly and has a low risk of metastasis, it can become locally invasive and destructive, causing significant tissue damage, particularly if diagnosis is delayed [3]. The diagnosis of BCC can be challenging, especially for less experienced dermatologists, because of the broad spectrum of benign and malignant lesions that can mimic this tumour [4]. In this context, new noninvasive diagnostic techniques, such as dermoscopy, reflectance confocal microscopy (RCM), line-field confocal optical coherence tomography (LC-OCT), and high-frequency ultrasounds (HFUSs), have gained considerable attention because they can assist physicians in the diagnosis of difficult lesions and reduce unnecessary excisions [5,6].

Dermoscopy is a cornerstone in dermatology [7], with training commonly incorporated into dermatology residency programs. In contrast, knowledge about other noninvasive skin imaging methods is still limited.

Unlike dermoscopy, imaging techniques such as RCM, LC-OCT, and HFUS enable virtual skin biopsies, allowing clinicians to discern histological features, although these methods are not directly equivalent to traditional histology [8]. Notably, the images produced by these techniques are greyscale, with only RCM and LC-OCT achieving cellular resolution [9]. RCM produces horizontal sections, which can be challenging for non-experts to interpret and correlate with vertical histological features [10]. In contrast, LC-OCT offers real-time imaging in both vertical and horizontal planes, combining the strengths of OCT and RCM [11] while addressing their respective limitations in spatial resolution, penetration depth, and image orientation. This comprehensive imaging capability makes LC-OCT more intuitive for comparison with histological images.

For these reasons and based on our experience, dermatologists may find LC-OCT images easier to interpret than earlier imaging devices. In addition, we hypothesize that this user-friendliness has likely contributed to the adoption of LC-OCT since its market introduction, especially in Europe.

Particularly, LC-OCT has demonstrated growing utility in clinical practice for BCC diagnosis when used by trained physicians [12,13]. Numerous studies have shown that LC-OCT not only improves the diagnostic accuracy of BCC but also aids in distinguishing BCC subtypes and monitoring the progression of lesions undergoing medical treatment [14].

To the best of our knowledge, although numerous studies have demonstrated the effectiveness of dermoscopy training programs in enhancing physicians’ diagnostic accuracy for pigmented skin lesions [15,16,17,18,19], research on the training required for LC-OCT use is still lacking. Moreover, no previous studies have assessed the relationship between training duration and performance in interpreting RCM, LC-OCT, or HFUS images.

It is generally believed that learning these techniques requires several months to years of practice. Considering the high cost of these devices, the extensive training time is often seen as a major obstacle to their widespread adoption within the dermatology community.

On this background, our study aimed to evaluate whether a single 1 h training program could help dermatology residents with no or low experience in LC-OCT improve their ability to differentiate BCC from other skin lesions using this imaging technique.

## 2. Materials and Methods

We conducted a descriptive study to assess the effect of a structured 1 h training course on the diagnostic performance of LC-OCT for the identification of BCC and non-BCC lesions.

### 2.1. Participants

Among dermatology residents of the Dermatology Department of the University of Siena, 8 dermatology residents with different levels of experience were selected. Four participants had no experience, and four participants were less experienced (two of them had <3 months of experience, while 2 participants had <1 year of experience with LC-OCT). A highly experienced dermatologist (E.C) with >10 years of experience in LC-OCT was included as the benchmark.

### 2.2. LC-OCT Course

**Figure 1:**
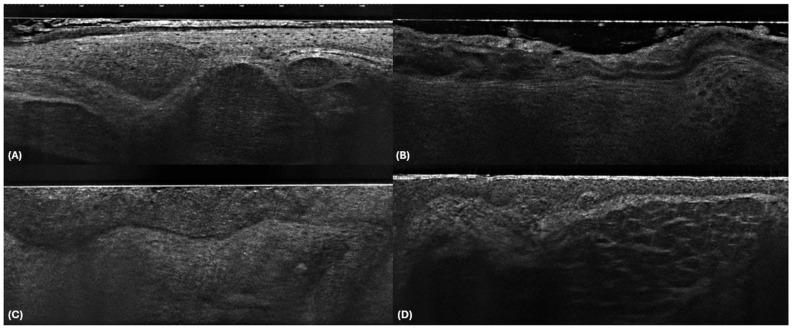
LC-OCT imaging of BCC (**A**), AK (**B**), SCC (**C**), and dermal nevus (**D**). In BCC (**A**), LC-OCT reveals characteristic features such as clefting, bright rim, and millefeuille pattern. AK (**B**) is marked by a disorganized epidermis with numerous atypical keratinocytes, an acanthotic epidermis, and a well-preserved, clearly outlined dermo-epidermal junction (DEJ). SCC (**C**) exhibits hyperkeratosis, a disorganized and acanthotic epidermis with abundant atypical keratinocytes, and a destroyed DEJ. In dermal nevus (**D**), the epidermis appears normal, while the dermis presents a regular wave pattern formed by hyperreflective stromal structures surrounding hyporeflective melanocytic dermal nests.

This training session was designed as a comprehensive, 1 h course focusing on the use of LC-OCT for the diagnosis of BCC and non-BCC lesions. The course included both a theoretical component and practical case-based instruction, which were designed to enhance participants’ understanding of LC-OCT and its application in diagnostics. The first 20 min of the training session were dedicated to a lecture-style presentation that introduced the principles of LC-OCT [20]. Participants were taught the fundamental physics of LC-OCT imaging, how the technology visualises subsurface skin structures at the cellular level, and how to interpret common patterns associated with BCC and non-BCC lesions (Figure 1) [20,21].

The training was provided by an expert in non-invasive skin imaging LC-OCT (E.C.) with the support of an expert in LC-OCT instruments (C.T; an engineer from the manufacturer of LC-OCT with more than 7 years of collaborations with dermatologists for the interpretation of LC-OCT images). The theoretical instruction focused on the specific morphological features visible in LC-OCT images that help identify BCC and distinguish it from its main imitators, such as dermal naevi, seborrhoeic keratosis, and sebaceous hyperplasia [1]. Key pitfalls and common diagnostic errors in LC-OCT image interpretation were also discussed. Table 1 presents the definitions used during the course.

### 2.3. Evaluation Phase

Participants’ performance was evaluated in three phases: before the training course, making diagnoses only with LC-OCT (pre-test), immediately after the training only with LC-OCT (post-test), and after the training, including dermoscopic images alongside LC-OCT (post-test with dermoscopy). The histopathology was not disclosed to the participants until the end of the entire examination. The cases were prepared by an external member (C.T, an engineer with more than 7 years of collaborations with dermatologists for the interpretation of LC-OCT images) that included 40 consecutive cases of LC-OCT from the database of the dermatology department of the University of Siena (Itay) comprising 20 BCC lesions and 20 non-BCC lesions. At each phase, participants evaluated the same 40 cases that were constituted by at least one LC-OCT 2D vertical image, one LC-OCT video, one LC-OCT 3D scan, and a dermoscopic image (the latter only in the third phase). Non-BCC included different lesions that could represent potential clinical and dermoscopic BCC imitators. Among the BCC lesions, 12 were superficial, 5 were invasive, and 3 were nodular. The non-BCC group included 4 seborrhoeic keratoses, 3 squamous cell carcinomas (SCC), 2 cases each of clear cell acanthomas, Bowen’s disease, lichenoid dermatitis, dermal naevi, and sebaceous hyperplasia, and one case each of wart, Leishmania, and cutaneous marginal zone B-cell lymphoma. All lesions were confirmed histopathologically. During the test, the participants were asked to classify each lesion as either BCC or non-BCC.

### 2.4. Statistical Analysis

Absolute frequencies and percentages were estimated for qualitative variables. The Fleiss Kappa was estimated to evaluate the concordance between dermatologists regarding diagnosis at each evaluation phase. Accuracy, sensitivity, and specificity and their 95% confidence intervals (95% CI) were estimated to compare diagnoses made by dermatologists with histological BCC diagnosis. A *p*-value < 0.05 was considered statistically significant. The analyses were performed using R version 4.3.1.

## 3. Results

Overall, 15 (38,5%) lesions were in the head/neck region, 13 (33.3%) were in the trunk, and 11 (28.8%) were in the extremities. The majority of BCC cases were located in the head/neck region (11, 57.9%), followed by the trunk (8, 42.1%). For the non-BCC cases, 11 (55.5%) were in the lower/upper extremities, 4 (20%) in the neck/head regions, and 5 (25%) in the trunk.

### 3.1. Evaluation Phase

**Figure 2:**
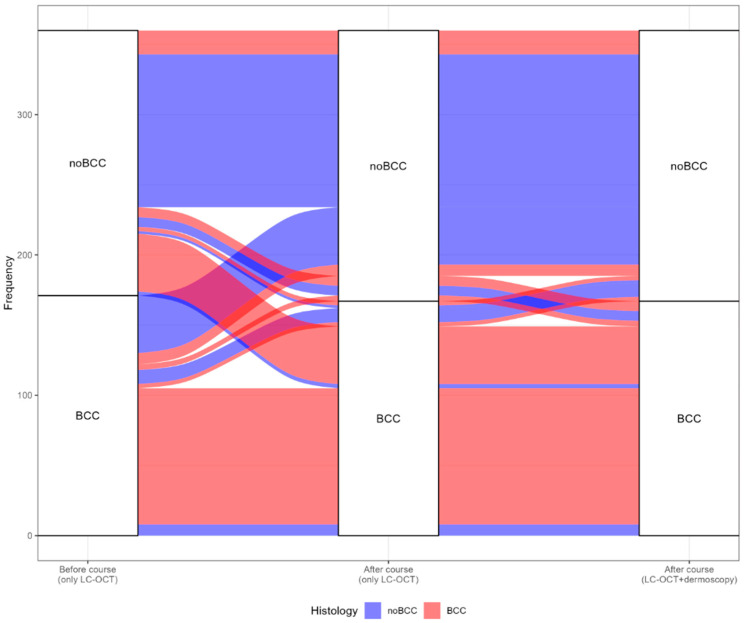
Diagnosis before and after the LC-OCT course and after the LC-OCT course with dermoscopy.

The trend of BCC evaluations performed by all participants before and after the training (both with and without dermoscopy) is shown in Figure 2. For the analyses reported in this paragraph, all 360 evaluations (40 cases × 9 participants) were considered.

The alluvial plot illustrates the changes in BCC diagnosis accuracy following LC-OCT training. Specifically, red bands represent histologically confirmed BCC cases, whereas blue bands denote histologically confirmed non-BCC cases. The upper section of the graph displayed images classified by participants as non-BCC, whereas the lower section showed images classified as BCC. Overall, the training course significantly reduced error rates, improving the correct classification of lesions. This improvement was evident in the red bands shifting from “non-BCC” in the pre-course evaluation to “BCC” in the post-course evaluation and from the blue band shifting from “BCC” to “non-BCC”. The graph also shows that the misclassification rate further decreased when dermoscopic images were incorporated into the assessment. However, the influence of dermoscopy was less pronounced than that of the LC-OCT course, as indicated by narrower blue bands ending in “non-BCC” and red bands ending in “BCC”. Notably, the plot revealed that one BCC and one non-BCC case were consistently misclassified throughout the evaluation. This evidence was confirmed by the agreement between participants at the three stages: 0.165 (*p* < 0.001) before the training course, and it increased to 0.598 (*p* < 0.001) after the course. An additional increase was observed when the test was repeated with dermoscopy, and the agreement reached 0.676 (*p* < 0.001). This emphasises greater appropriateness and concordance in responses after the course.

### 3.2. Evaluation Phase According to Participant Experiences

Table 2 shows the results of the pre- and post-training tests, stratified by the level of participant’s experience in LC-OCT. The total number of evaluations considered for the non-experience analysis was 160 (40 cases × 4 participants); it was 160 for the low-experienced (40 cases × 4 participants) and 40 for the experienced (40 cases × 1 participants).

For dermatology residents not experienced in LC-OCT before training, an incorrect BCC diagnosis was made in more than 50% of cases. Specifically, 42 (52.5%) BCC cases were incorrectly classified as non-BCC, and 41 (51.2%) non-BCC cases were incorrectly classified as BCC based on only LC-OCT images. However, after the training phase, their performance in evaluating BCC at LC-OCT increased significantly, showing a 20% decrease in false negative rates and a 35% decrease in false positive rates. When dermoscopic images were added to the test, a higher number of correct BCC diagnoses were detected (an additional 5% in both false positives and false negatives rates). In addition, the reduction in the error rate between the pre-training and post-training periods was confirmed by the accuracy, sensitivity, and specificity (Table 3).

Among the four inexperienced readers, the overall diagnostic rate increased significantly after training in the diagnostic method from 77/160 (0.48; CI 95%: 0.40–0.56) to 121/160 (0.76; CI 95%: 0.68–0.82), a difference of 28%. After the third test, which also included the dermoscopy images, there was an additional increase in the overall diagnostic rate, rising to 0.81 (CI 95%: 0.74–0.86), with an increase of 5% compared with the post-training test that only included LC-OCT images.

Considering dermatology residents with low experience with LC-OCT, an improvement in diagnostic performance was observed with training. Before the training period, these participants demonstrated better performance compared to the four inexperienced readers, with 22.5% fewer false negatives and 31.2% fewer false positives compared to the no-experience group.

After the training period for the two participants with less than 3 months of experience, the number of BCC cases incorrectly classified as non-BCC decreased from 30% (24/80) to 11.2% (9/80), representing a difference of 18.8%. Similarly, the non-BCC cases incorrectly classified as BCC decreased from 20% (16/80) to 11.2%, representing a reduction of 8.8%. In the test that included the dermoscopic images, there was only a minimal reduction in the false negative rate (by 1.2%, and no reduction in the false positive rate was observed). The improvement in the diagnostic rate can also be seen in the two participants with less than 3 months of experience, from 120/170 (0.75, CI 95%: 0.68–0.81) to 142/170 (0.89 CI 95%: 0.83–0.93), a difference of 14%. For these cases, the accuracy did not show further improvement in the test that included the dermoscopy images.

Lastly, the dermatologist with several years of experience with LC-OCT had high sensitivity and specificity for diagnosing BCC when evaluating LC-OCT images. Both the false-positive and false-negative rates were only 5% (1/20) in the evaluation where only the LC-OCT was shown. When the dermoscopic images were included in the evaluation, no false-positive results were observed.

## 4. Discussion

Training in advanced diagnostic tools, such as LC-OCT and dermoscopy, is a key factor in improving diagnostic outcomes among dermatologists. For the first time, our study focused on the impact of LC-OCT training on the diagnostic accuracy of BCC, showing that the diagnostic performance of dermatology residents in BCC detection, particularly in those who were inexperienced, significantly improved LC-OCT post-training, with significant reductions in both false positives and false negatives.

Interestingly, numerous studies have investigated the impact of structured dermoscopy training on diagnostic accuracy, demonstrating that such training improves the ability of healthcare professionals, including those with limited experience, to diagnose skin cancer [15,17,22,23,24].

First, Augustsson et al. [15] assessed the impact of a 1-day dermoscopy training course on the diagnostic accuracy of general practitioners. Participants who underwent training demonstrated significant improvement in their test scores immediately after the course, which was maintained after 6 months. In contrast, the control group showed only marginal, nonsignificant improvement over the same period. Similarly, Barten et al. [24] demonstrated that a structured training program could serve as a crucial resource for clinicians, including both dermatologists and non-dermatologists, particularly in non-specialist settings, by enhancing early detection and accurate classification of skin cancer lesions.

In our study, we found that, before the training, over half of the BCC cases were misclassified as non-BCC (52.5%), and non-BCC cases were frequently mistaken for BCC (51.2%) by dermatologists who were inexperienced in LC-OCT. However, LC-OCT training significantly improved diagnostic accuracy, reducing false negatives by 20% and false positives by 35%. This result clearly reflects the lack of familiarity with LC-OCT features, which contributes to the high rate of misclassification in BCC detection. Given that LC-OCT images are displayed in greyscale, their initial interpretation can be challenging for those encountering them for the first time. Interestingly, in this group, the incorporation of dermoscopic images further improved the performance, reducing error rates by an additional 5%. These findings can likely be explained by the fact that dermoscopy is a technique commonly used and well-established among dermatology residents who tend to be more confident in their ability to interpret dermoscopic images compared to other imaging modalities. As a result, the addition of dermoscopic images to the diagnostic process resulted in a notable improvement in diagnostic accuracy.

Overall, diagnostic accuracy increased from 48% pre-training to 76% post-training and reached 81% when dermoscopic images were included, highlighting the effectiveness of structured training combined with multimodal imaging in low-experienced dermatologists. This may suggest that clinicians who are trained in both LC-OCT and dermoscopy are better able to understand lesion morphology and make more informed decisions regarding the necessity for further investigation or treatment. This combined expertise enhances their diagnostic abilities and ultimately improves patient outcomes. As demonstrated with RCM, integrating dermoscopy with advanced imaging techniques can improve diagnostic accuracy [25]. Similarly, the use of dermoscopy, which is now integrated into the LC-OCT device, could further strengthen its diagnostic potential.

**Figure 3:**
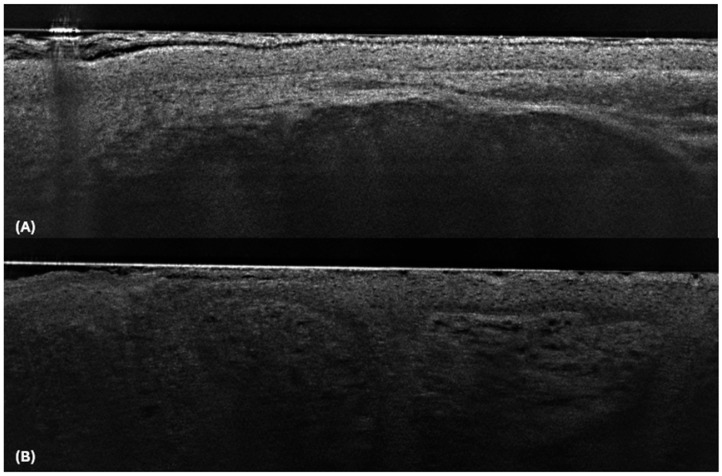
LC-OCT image of Sebaceous hyperplasia (**A**): A well-defined dark lobule is visible in the dermis. Unlike in BCC, there is no millefeuille pattern within the lobule, and hyperreflective cells are present only in the upper portion. The deeper layers exhibit signal blockage due to the fat content within the cells. LC-OCT image of BCC (**B**): In this case, the tumour lobules were not surrounded by clefting, and the surrounding stroma did not appear particularly bright.

We also observed an improvement in diagnostic performance among low-experienced dermatology residents following LC-OCT training. As expected, before training, the participants demonstrated better accuracy than inexperienced readers, with 22.5% fewer false negatives and 31.2% fewer false positives compared to the no-experience group. After training, the misclassification of BCC as non-BCC decreased significantly from 30% to 11.2%, and the misclassification of not BCC as BCC decreased from 20% to 11.2%. However, when dermoscopic images were added, there was only a slight reduction in false negatives (1.2%) and no change in false positives. Diagnostic accuracy improved overall, with participants’ scores increasing from 0.75 to 0.89; however, no further enhancement was observed with the addition of dermoscopy images. This finding is consistent with the results reported by Barten et al. [24], in which clinicians with more dermoscopy experience exhibited smaller improvements compared with novices. For highly experienced users, the added value of incorporating dermoscopic images may be minimal because they are likely already well-acquainted with the morphological characteristics of BCC and non-BCC lesions through prior clinical experience. Notably, the plot revealed that one BCC and one non-BCC case were consistently misclassified throughout the evaluation (Figure 3). In this BCC case, the usual distinguishing features (clefting and bright stroma) were barely recognizable, while in sebaceous hyperplasia, a well-defined lobule was present, resembling BCC.

The main strength of our study is that after just one hour of training, we observed improvement in diagnostic performance, especially among inexperienced dermatologists. This suggests that LC-OCT may represent a “user-friendly” tool that can enhance diagnostic ability and performance for detecting skin cancers, particularly BCC. In this context, early and accurate diagnosis of BCC is crucial because it can prevent disease progression and reduce the need for more invasive treatment.

Although our study provides valuable insights into the impact of training and the use of dermoscopy on diagnostic accuracy, several limitations must be acknowledged. First, the relatively small sample size may limit the generalizability of the results. Thus, future studies should aim to replicate these findings in larger and more diverse populations to ensure that improvements in diagnostic accuracy can be broadly applied across different clinical settings. Second, this study did not include long-term follow-up to assess the retention of diagnostic skills over time. As previously mentioned, ensuring the sustainability of the gains achieved through training is a critical issue that warrants further investigation. Another possible consideration is that training only focused on image interpretation and not on image acquisition, which is also part of the imaging examination. Lastly, the study focused primarily on BCC, and a limited number of BCC imitators could be included in the differential diagnosis.

## 5. Conclusions

In conclusion, this study demonstrated the significant impact of structured training on LC-OCT and the use of dermoscopy coupled with LC-OCT on improving the diagnostic accuracy of skin lesions. The results highlight the importance of tailored training programs for enhancing clinicians’ ability to accurately classify BCC and non-BCC lesions, particularly for less experienced readers. The addition of dermoscopic images provided further improvements in diagnostic performance, particularly for novice users, although the benefits were less pronounced for experienced clinicians. These findings align perfectly with previous research and suggest that the integration of advanced imaging tools into routine clinical practice, particularly in non-specialist settings, can lead to better diagnostic outcomes and improved patient care. However, further studies should be performed to evaluate the long-term effects of training on diagnostic accuracy.

## Figures and Tables

**Table 1:** LC-OCT definitions and common diagnostic pitfalls of different skin conditions.

**BCC**	**Clefting** The clefting corresponds to the middle dark rim surrounding the lobule. It probably corresponds to peritumoral mucin deposition.
	**Bright rim** The outer rim surrounding the lobule characterised by a brighter colour than the stroma. This appearance is probably due to compression and/or alteration (mass effect) of the collagen fibres of the stroma by the tumour islands.
	**Millefeuille pattern** The cores of BCC lobules correspond to dense cellularity oriented along the same axis, composed of basaloid cells.
**BCC subtypes**	
Superficial	Lobules are connected to the epidermis. The lobules tend to be small and usually have a hemispheric shape. The dark rim is particularly visible in the deeper portion of the lobules. Lobules of superficial BCC less frequently display classic triads because the bright rim is often absent because of less involvement of the surrounding stroma.
Nodular	Lobules are separated from the epidermis. Lobules are large with a roundish shape and clear borders. Stroma is very bright, and its rim can be seen above the lobules. The overlying epidermis might be thin due to the tumour pressure.
Infiltrating	Lobules have a complex shape with branches infiltrating the dermis. Lobules mostly lie in the dermis, although some of them can be connected to the epidermis. The stoma is altered by the lobules of the branches.
**Skin lesions other than BCC**	
**Actinic keratosis**	The epidermis is disorganised with several atypical keratinocytes. The epidermis can be atrophic or acanthotic, but the DEJ is always conserved and outlined. A few small tumour buddings of the epidermis into the dermis can be seen. Elastosis or inflammation can be observed in the dermis.
**SCC in situ/Bowen disease**	The epidermis is disorganised and acanthotic, with numerous atypical keratinocytes. The DEJ is not always outlined, and tumour budding of keratinocytes invades the dermis. Elongated rete ridges and superficial glomerular vessels can present due to epidermal papillomatosis, particularly in Bowen’s disease. If not out of the field of view (FOV), solar elastosis or inflammation can occur in the dermis.
**SCC**	The stratum corneum is particularly thick (hyperkeratosis). Sometimes, ulceration and crusts can be observed. The epidermis is disorganised and acanthotic, with numerous atypical keratinocytes. The DEJ is destroyed by deep and large tumour buddings and board stands invading the dermis. If not out of the field of view (FOV), solar elastosis or inflammation can occur in the dermis.
**Dermal Naevus**	Presence of a regular wave pattern in the dermis created by hyperreflective structures corresponding to the stroma surrounding hyporeflective melanocytic dermal nests. The epidermis is normal. In some cases, hyperreflective cells can be seen inside dermal nests. The absence of the Millefeuille pattern inside melanocytic nests allows for the differentiation of dermal naevus from BCC.
**Seborrhoeic keratosis**	Epidermal invaginations and intraepidermal pseudocysts filled with hyperreflective keratin (onion shape). Acanthosis and elongated rete ridges.
**Inflammatory Skin conditions**	The epidermis can be acanthotic. The DEJ can be blurred by a lichenoid inflammatory infiltrate or wavy by papillomatosis. Bright spots corresponding to inflammatory cells may be present in the dermis and vessels, which are often dilated.
**Sebaceous hyperplasia**	Dark lobules in the dermis. Lobules may present as cleft and bright rims. However, differentiation with BCC is possible based on the following criteria: Lobules are connected to hair follicles. There is no millefeuille pattern inside the lobules; instead, in the upper part of the lobules, large hyperreflective r cells corresponding to sebaceous cells are visible. Sebaceous cells are not visible in the mid- and lower parts of the lobules because their content blocks signal transmission. The epidermis is normal and is not affected by underlying sebaceous hyperplasia.

Legend: BCC, basal cell carcinoma, SCC, squamous cell carcinoma.

**Table 2:** Classification of BCC and non-BCC lesions by experience level and training stage.

		Non-Experienced		Low-Experienced		Experienced	
		Non-BCC	BCC	Non-BCC	BCC	Non-BCC	BCC
**Pre Training**	Non-BCC	39 (48.8%)	42 (52.5%)	64 (80.0%)	24 (30.0%)	19 (95%)	1 (5%)
	BCC	41 (51.2%)	38 (47.5%)	16 (20.0%)	56 (70.0%)	1 (5%)	19 (95%)
**Post Training**	Non-BCC	67 (83.8%)	26 (32.5%)	71 (88.8%)	9 (11.2%)	19 (95%)	1 (5%)
	BCC	13 (16.2%)	54 (67.5%)	9 (11.2%)	71 (88.8%)	1 (5%)	19 (95%)
**Post Training + dermoscopy**	Non-BCC	71 (88.8%)	22 (27.5%)	71 (88.8%)	8 (10.0%)	20 (100.0%)	1 (5%)
	BCC	9 (11.2%)	58 (72.5%)	9 (11.2%)	72 (90.0%)	0 (0.0%)	19 (95%)

**Table 3:** Sensitivity, specificity, and accuracy of BCC classification according to experience level and training stage.

		Non-Experienced	Low-Experienced	Experienced
**Pre-Training**	SE	0.47 (0.36, 0.59)	0.70 (0.59, 0.80)	-
	SP	0.49 (0.37, 0.60)	0.80 (0.70, 0.88)	-
	Accuracy	0.48 (0.40, 0.56)	0.75 (0.68, 0.81)	-
**Post-Training**	SE	0.68 (0.56, 0.78)	0.89 (0.80, 0.95)	0.95 (0.75, 1.00)
	SP	0.84 (0.74, 0.91)	0.89 (0.80, 0.95)	0.95 (0.75, 1.00)
	Accuracy	0.76 (0.68, 0.82)	0.89 (0.83, 0.93)	0.95 (0.83, 0.99)
**Post-Training + dermoscopy**	SE	0.72 (0.61, 0.82)	0.90 (0.81, 0.96)	0.95 (0.75, 1.00)
	SP	0.89 (0.80, 0.95)	0.89 (0.80, 0.95)	1.00 (0.83, 1.00)
	ACC	0.81 (0.74, 0.86)	0.89 (0.84, 0.94)	0.97 (0.87, 1.00)

Legend: SE, sensitivity; SP, specificity.

## Data Availability

The data presented in this study are available upon request from the corresponding author.
